# Validation study of the Polish version of the Evidence-Based Practice Profile Questionnaire

**DOI:** 10.1186/s12909-017-0877-4

**Published:** 2017-02-10

**Authors:** Mariusz Panczyk, Jarosława Belowska, Aleksander Zarzeka, Łukasz Samoliński, Halina Żmuda-Trzebiatowska, Joanna Gotlib

**Affiliations:** 1 Division of Teaching and Outcomes of Education, Faculty of Health Science, Medical University of Warsaw, Żwirki i Wigury 61, 02-091 Warsaw, Poland; 2 Division of Clinical Nursing, Faculty of Health Science, Medical University of Warsaw, Erazma Ciołka 27, 01-445 Warsaw, Poland; 3 Centre of Postgraduate Education for Nurses and Midwives in Warsaw, Adolfa Pawińskiego 5A, 02-106 Warsaw, Poland

**Keywords:** Psychometrics, Questionnaires, Reproducibility of results, Health knowledge attitudes practice, Evidence-based practice, Patient safety

## Abstract

**Background:**

Decisions about patient care in clinical practice should be made based on proven scientific evidence of efficacy and safety (i.e., evidence-based practice [EBP]). Currently, there are no available tools in Poland for assessing the knowledge and attitudes of specialists in health sciences towards EBP. Therefore, by validating the Polish version of the original English Evidence-Based Practice Profile Questionnaire (EBP^2^Q), we may provide an appropriate instrument for assessing EBP.

**Methods:**

The validation group consisted of 1,362 people, including nurses and midwives taking the specialization exam, second-degree students in nursing/midwifery, and staff of selected municipal and clinical hospitals in Warsaw, Pruszkow, and Chelm. The study was conducted from March to June 2014. The following psychometric properties of the EBP^2^Q were assessed: reliability (Cronbach’s alpha coefficient, and test-retest), validity (exploratory factor analysis, Spearman’s r correlation coefficient, and assessment of inter-group differences), as well as unidimensionality of domains (principal component analysis).

**Results:**

All domains of the EBP^2^Q were characterized by high reliability (Cronbach’s alpha ranging from 0.800 to 0.972). The Polish version showed a strong similarity of factor structure with the original English EBP^2^Q, indicating that the condition for theoretical validity is fulfilled. Maintenance of the theoretical and discriminative validity and unidimensionality of five domains of the EBP^2^Q was confirmed.

**Conclusions:**

The Polish version of the EBP^2^Q is comparable in terms of psychometry to the original English version. This questionnaire can be used to assess knowledge, attitudes, and skills concerning EBP among students and practicing professional nurses and midwives. The future validation of the EBP^2^Q in other groups of specialists in health sciences may increase the scope of applicability of this tool.

**Electronic supplementary material:**

The online version of this article (doi:10.1186/s12909-017-0877-4) contains supplementary material, which is available to authorized users.

## Background

The paradigm of Evidence-Based Practice (EBP) is based on an assumption that clinical decisions in patient care are an outcome of patients’ values, clinical circumstances and the best research evidence [1]. It is assumed that through such medical practice patients receive the best possible care that is not largely backed by tradition and convictions but stems from the most up-to-date research (the most valid and reliable scientific evidence) [1, 2]. In addition, inclusion of patients’ values in the decision-making process leads to increased acceptance of proposed treatment by the patient [3]. EBP principles, when followed by nurses, are believed to improve the quality of patient care, aid their professional development and help them accept responsibility for decisions made [3]. Further, application of EBP is likely to reduce costs of medical care by elimination of ineffective and risky medical procedures [4].

As a result of the above benefits arising from application of EBP principles, there is an increased focus on formation of EBP-related attitudes and skills in under- and post-graduate education [5, 6]. In order to make decisions based on the paradigm of the EBP, nurses need, on the one hand, to accept such a method of medical practice (a positive attitude), and on the other, they need to have the necessary skills and expertise required for a critical analysis of available scientific evidence [7]. Although the ability to use research results is an important part of the development of nurses and other related professions, there are numerous barriers to the implementation of EBP. One major obstacle is the insufficient competence of nursing staff in the practical aspects of EBP [8]. According to Solomons and Spross [9], additional training concerning searching, evaluation, and practical use of available scientific evidence is necessary at all levels of nurse education. Therefore, both professional studies and postgraduate education should involve training nurses in the methodology of research, as well as teaching them the skills necessary to use EBP at work [10, 11].

Implementation of appropriate educational solutions that are tailored to the individual needs of a particular group requires a preliminary assessment of competencies with the use of standardized questionnaires [12]. Most tools used to assess competence in EBP focus on individual elements of knowledge and skills that are necessary to implement care based on scientific evidence. In addition, questionnaires investigating the presence of potential barriers to implementation of the concept of EBP in the workplace have been developed, which evaluate the attitudes and behavior of individual members of the therapeutic team [13, 14]. Despite this, an overview of the Polish scientific literature concerning EBP published in 2014 indicated that there are currently no available studies on the assessment of knowledge and attitudes of specialists in health sciences towards EBP [15]. In addition, there are unexplored areas, such as the level of competence in the EBP or the existing barriers in the implementation of the concept of EBP in Polish health care facilities. Indeed, no appropriate tools of proven psychometric properties have been developed in Poland that could be used to study key aspects of EBP functioning in individual groups of health sciences professionals.

Globally, a few types of questionnaires for assessing EBP are available, including: the *Evidence-Based Practice Profile Questionnaire* (EBP^2^Q) [16], the *Evidence-Based Practice Questionnaire for Nurses* [17, 18], the *Evidence-Based Nursing Attitude Questionnaire* [19], the *Evidence-Based Practice Attitude Scale* [13], the *Developing Evidence-Based Practice Questionnaire* [20], and the *Evidence Based Practice Evaluation Competence Questionnaire* [21]. Although the range of available research tools is extensive, according to Shaneyfelt et al. [22], further development, adaptation, and evaluation of new questionnaires, adapted to local conditions and specifics of work in the given country are needed. Therefore, we adapted one of the more universal questionnaires, the EBP^2^Q [16]. The EBP^2^Q was developed at the University of South Australia by a team led by Maureen McEvoy, and was validated on 526 people (consisting of students, academic teachers, and practitioners) [16]. Apart from its good psychometric parameters, an additional advantage of the EBP^2^Q is its application to self-assessment of EBP competences by students, lecturers, and practitioners [16]. It can also assess different aspects of EBP by selecting individual parts (domains) of the questionnaire [16]. The purpose of this study was to validate the psychometric properties of a Polish version of the validated English EBP^2^Q.

## Methods

### Evidence-based practice profile questionnaire

The original EBP^2^Q consists of 58 statements (all based on a five-point Likert scale), allowing the assessment of five domains (Relevance, Terminology, Confidence, Practice, and Sympathy) by the respondent. It is supplemented with additional questions necessary for the sociodemographic characteristics of respondents. In addition, the EBP^2^Q contains 16 statements, which have not been assigned to any of the five domains in the course of validation (Table 1).

**Table 1:** Structure of the EBP^2^Q with the separate domains and contained statements^a^

Domain	Item numbers	Description
I. Relevance	1–14 (14 items)	Attitude towards expanding own competence in the Evidence-Based Practice, expressed on a scale from 1 to 5 (1 – not at all true; 5 – very true)
II. Sympathy	15–21 (7 items)	Attitude towards selected aspects of the Evidence-Based Practice in work, assessed by respondents on a scale from 1 to 5 (1 – strongly disagree; 5 – strongly agree)
III. Terminology	22–38 (17 items)	The level of knowledge about the terminology related to scientific research; given terms and issues were rated on a scale from 1 to 5 (1 – never heard the term; 5 – understand and could explain to others)
IV. Practice	39–47 (9 items)	Frequency of use of individual elements of Evidence-Based Practice in daily clinical work, assessed on a scale from 1 to 5 (1 – never; 5 – daily)
V. Confidence	48–58 (11 items)	Confidence in skills related to Evidence-Based Practice rated on a scale from 1 to 5 (1 – not at all confident; 5 – very confident)
VI. Non-domain items	59–74 (16 items)	Other aspects of Evidence-Based Practice, expressed on a scale from 1 to 5 (1 – strongly disagree; 5 – strongly agree)
VII. Demographics	-----	Selected sociodemographic variables

^a^all items based on a five-point Likert scale

The original English version of the EBP^2^Q is available at: http://www.biomedcentral.com/content/supplementary/1472-6920-11-100-s1.pdf

The authors of the present study obtained the agreement of the authors of the original EBP^2^Q to use the tool in the studies conducted by the Medical University of Warsaw. The EBP^2^Q in the form proposed by McEvoy et al. [16] was translated into Polish by two independent translators. Review and comparison of the two translations by the authors and a bilingual expert in nursing demonstrated their close similarity. The agreed version of the Polish translation was not subjected to reverse translation, and therefore, the final version of the questionnaire was not tested on bilingual people. The form of the agreed Polish version of the EBP^2^Q is included in Additional file 1 *.*

### Data collection

The study involved 1,362 people, including 1,195 (87.7%) women, 42 (3.1%) men, and 125 persons (9.2%) who did not respond to the question regarding sex. The average age was 39.4 years (min. 20, max. 69, SD = 10.32, CV = 26.2%). The study group consisted of three subgroups: (1) nurses and midwives taking the state specialization exams organized by the Centre of Postgraduate Education for Nurses and Midwives in the spring session in 2014 (*N* = 596); (2) second-degree students of nursing and midwifery at the Medical University of Warsaw and the Higher Vocational School in Kalisz (*N* = 462); and (3) nurses working in municipal and clinical hospitals in Warsaw, Pruszkow, and Chelm (*N* = 304). The study was conducted from March to June 2014. Participation in the study was voluntary and anonymous. Results were collected using an auditorium questionnaire, in which respondents were gathered in a single room at the hospital to complete self-administered questionnaires and the completed questionnaires were subsequently pooled.

### Methods of psychometric analysis

EBP^2^Q validation was carried out in reliance on recommendations proposed by Downing [23] and Sullivan [24]. To evaluate the psychometric properties of the EBP^2^Q, an analysis of reliability, validity, and unidimensionality of the five selected domains was performed. To assess the reliability of the EBP^2^Q, analysis of the internal consistency of the given domain was applied, using the formula proposed by Cronbach [25]. According to Nunnally’s criterion [26], a reliability threshold level with a Cronbach’s alpha greater than 0.70 was considered acceptable. To assess the intrascale compatibility of particular statements, the correlation matrix was determined, and the value of *r* > 0.30 was assumed the criterion for consistency [27].

The test-retest reliability was assessed in a group of 160 students who were retested using the EBP^2^Q following a 2-week interval. Absolute stability was measured by calculating the weighted kappa coefficient and the intra-class correlation coefficient (ICC) that determine the level of consistency of answers given between the first and the second measurement [24]. To establish a correct test-retest analysis, the assumption of equal means in two measurements was checked using the *t* test [27]. The criteria for the assessment of the test-retest reliability were analogous to those used for validation of the English version of the EBP^2^Q [16].

The validity of EBP^2^Q was assessed using three different analytical approaches. First, estimation of theoretical validity, also known as the internal validity, was performed by exploratory factor analysis. In particular, we explored whether the factor structure of the EBP^2^Q consists of five domains. Two different factor analysis criteria were used: (1) the Kaiser criterion, based on the number of factors with eigenvalues over one [28]; and (2) the Cattell’s criterion, based on extracting the number of factors from the steep curve of the scree plot [29]. Both criteria were used as the Kaiser criterion can result in overestimating the number of factors (especially with the large number of items in the EBP^2^Q), while the more conservative approach based on the scree plot gives more reliable results [30]. The fulfillment of assumptions for factor analysis was evaluated, the degree of homogeneity of variance was estimated, the determinant of the correlation matrix and the Kaiser-Meyer-Olkin (KMO) measure of sampling were determined, and the Bartlett’s test of sphericity was performed. Second, estimation of theoretical validity, based on the determination of the degree of correlation between the selected domains of the questionnaire, was assessed. To verify the above assumption, the Spearman’s rank correlation coefficient was calculated. Third, validity was estimated based on assessment of inter-group differences (discriminative validity). To do so, a comparative analysis of two groups of students was performed. One group took an e-learning EBP course (*N* = 119) while the control group did not participate in additional EBP classes (*N* = 207). A detailed training course had been previously published by Panczyk et al. [31]. The *t* test was used to compare the groups and Cohen’s *d* was determined as a measurement of the effect of differences between means. The following criteria were assumed to assess the measured effect size: very strong ≥0.80, strong 0.50–0.79, average 0.49–0.20, and poor <0.2 [32].

The analysis of unidimensionality of each EBP^2^Q domain was performed using principal component analysis. A domain was considered unidimensional if it met the Kaiser criterion [28] (i.e., designated eigenvalues exceeded the value of 1 only once), and if the degree of explanation of variability (i.e., using indicator variables via the first main component) exceeded 40%.

All statistical calculations were performed using the statistical package IBM® SPSS® Statistics, version 23. For all analyses, a P-level of < 0.05 was considered statistically significant.

## Results

### Analysis of reliability

All five domains constituting the EBP^2^Q met Nunnally’s criterion. Moreover, the mean correlations between individual statements for the corresponding domains were higher than the recommended value of 0.30. The analysis of each domain, in terms of changes in the value of Cronbach’s alpha coefficient after the removal of individual statements, showed that only the elimination of one domain item (No. 15) caused a slight increase in the value of the coefficient by 0.016 (see Additional file 2, “Reliability”). Details of the results of reliability analysis are presented in Table 2.

**Table 2:** Reliability of the Polish version of the EBP^2^Q: Cronbach’s alpha coefficient

Domain	Item numbers	Cronbach’s alpha	Mean correlation
I. Relevance	1–14 (14 items)	0.937	0.698
II. Sympathy	15–21 (7 items)	0.798	0.532
III. Terminology	22–38 (17 items)	0.971	0.801
IV. Practice	39–47 (9 items)	0.923	0.725
V. Confidence	48–58 (11 items)	0.940	0.744

The assessment of test-retest reliability shows a good stability of scales in individual domains of the EBP^2^Q. The assumption concerning the equality of the means for the repeated measurements was fulfilled, and the value ranges of weighted kappas and ICCs for the items were satisfactory (Table 3).

**Table 3:** Test-retest reliability of the questionnaire

Domain	Range of weighted kappas for items in each domain^a^	Range of ICCs for items in each domain^b^	Domain ICCs	Mean difference	0.95 CI for mean difference	*P*-value^^^
I. Relevance	0.35–0.74	0.62–0.89	0.94	0.42	−1.90–1.06	0.577
II. Sympathy	0.22–0.52	0.22–0.67	0.68	0.37	−1.24–0.50	0.405
III. Terminology	0.35–0.80	0.59–0.91	0.97	1.50	−3.28–0.28	0.099
IV. Practice	0.26–0.54	0.43–0.70	0.92	0.84	−2.04–0.36	0.169
V. Confidence	0.25–0.70	0.52–0.85	0.95	0.78	−1.77–0.22	0.125

^a^Kappa values ≥0.80 were taken to represent excellent agreement, 0.60–0.79 substantial agreement and 0.40–0.59 moderate agreement

^b^For ICCs, values more than 0.75 indicated good reliability and less than 0.75 poor to moderate reliability

^^^ *t* test to compare mean differences in two measurements

### Analysis of theoretical validity

Before we estimated theoretical validity using factor analysis, we verified the fulfillment of the assumptions for this method. No statements showed a standard deviation of zero, and we confirmed the presence of homoscedasticity (Levene’s test for equality of variances, *P* > 0.05). In addition, the value of the determinant of the correlation matrix was close to zero (1.4∙10^−28^). Furthermore, the condition for sphericity was fulfilled, as it was found that a correlation coefficients matrix is not an identity matrix (Bartlett’s test of sphericity, *P* < 0.0001). The last condition of factor analysis was evaluated by the KMO test to assess the expected reduction rate. Adequacy of sampling (KMO index) was 0.949, which fulfills the assumptions for this parameter (KMO > 0.5).

**Fig. 1:**
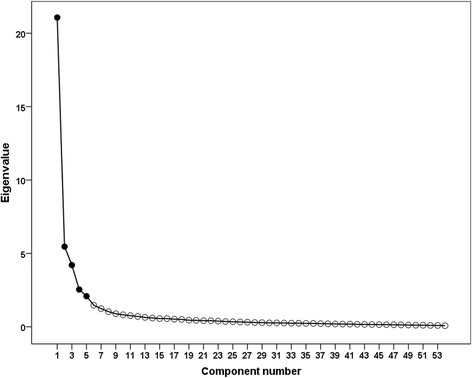
Scree plot illustrating the components extracted from the data*. * The number of components retained is five, as indicated by the change in shape of the plot after the fifth component. The labels indicate the percentage of variance explained by each factor

In the first attempt of exploratory factor analysis, 58 statements were distributed in accordance with the Kaiser criterion in eight domains, which was not consistent with the concept of the distribution of statements into five domains. However, interpretation of the scree plot (Cattell’s criterion) indicated five domains (Fig. 1). In the next analysis, a 5-factor solution was imposed in accordance with the theoretical assumptions. It turned out that the variables located in five factors explained more than 63% of the total variance.

To facilitate the interpretation of the obtained non-orthogonal solution, direct oblimin rotation of raw factor loadings was performed. The resulting solution largely confirmed the proposed structure of the EBP^2^Q. All domains proved to be theoretically valid. Only item No. 15 from domain II (Sympathy), did not load as expected with other statements of this domain. The result of the rotation indicates that this item belongs to domain I (Relevance) rather than domain II (Sympathy). Detailed summary of the results of the factor analysis for the direct oblimin rotation of loadings are included in Additional file 2, “Factor analysis”.

Theoretical validity of the EBP^2^Q was also evaluated by calculating the Spearman’s rank correlation coefficients based on the sum of the scores calculated for each of the domains. For domain II (Sympathy), a weak correlation with domain I (Relevance, *r*_s_ = 0.13) was found. No significant positive correlation with other domains of the EBP^2^Q was found (Table 4). Particularly good correlation coefficients were observed for domain IV with domain V and for domain III with domain IV. This indicates high criterion validity, which can be expressed as a positive correlation between the “Practice” and “Confidence” (*r*_s_ = 0.60) and “Practice” and “Terminology” (*r*_s_ = 0.51).

**Table 4:** Correlation between the results for the individual domains: Spearman’s rank correlation coefficients

	Domain				
	I	II	III	IV	V
I. Relevance	----	0.13	0.49	0.45	0.49
II. Sympathy	0.13	----	−0.02*	−0.02*	0.05*
III. Terminology	0.49	−0.02*	----	0.51	0.47
IV. Practice	0.45	−0.02*	0.51	----	0.60
V. Confidence	0.49	0.05*	0.47	0.60	----

*Correlation coefficients not statistically significant (*P* > 0.05)

### Analysis of discriminative validity

Within three domains (Relevance, Sympathy, and Terminology) the mean score of the students participating in the EBP training was significantly higher compared with that of the control group students. EBP training had a small to medium effect size on scores within these three domains (Cohen’s *d* 0.29–0.43). In the case of domain V (Confidence), however, the difference was on the border of statistical significance and the effect size was small (*P* = 0.054, *d* = 0.22). No significant effect was noted for the students’ score in domain IV (Practice, *d* = 0.01). The results of the validity assessment using the inter-group differences method are presented in Table 5.

**Table 5:** Comparison of scores obtained in EBP^2^Q - 12 h e-learning course group vs. control group

Domain	No formal training (*N* = 207) mean (SD)	12 h course (*n* = 119) mean (SD)	*P*-value*	Effect size Cohen’s *d*
I. Relevance	47.6 (10.86)	51.9 (9.64)	0.001	0.43
II. Sympathy	20.7 (3.77)	22.0 (4.29)	0.005	0.32
III. Terminology	38.7 (17.28)	43.4 (14.71)	0.010	0.29
IV. Practice	20.2 (9.08)	20.3 (7.38)	0.925	0.01
V. Confidence	37.0 (8.40)	38.8 (7.63)	0.054	0.22

*t test to compare mean values

### Assessment of unidimensionality of domains

Based on a determined structure of the EBP^2^Q, the unidimensionality of domains was evaluated. That is, we assessed whether each domain can be considered unidimensional. Using principal component analysis, we verified the eigenvalues and the share of variance explained by the first factor (Table 6). For domains III, IV, and V only one eigenvalue was greater than 1, which, according to Kaiser criterion, demonstrated unidimensionality for each of them. For domains I and II, the second factor indeed exceeded the limit value of criterion, but the percentage of explained variance was above the expected threshold of 40%.

**Table 6:** The share of variance explained by first principal component analysis

Domain	Kaiser criterion^a^	The share of variance explained by the first principal component (%)
I. Relevance	7.82; 1.91	55.84
II. Sympathy	3.26; 1.13	46.57
III. Terminology	11.63; 0.95	68.41
IV. Practice	5.61; 0.72	62.30
V. Confidence	6.95; 1.00	63.18

^a^eigenvalue of 1 and 2 factor, respectively

## Discussion

Although the concept of EBP has been known for decades, this concept is not widely known in the Polish nursing and obstetric environment [15]. With the development of nursing research, and the growing role of the results of these studies in the decision-making process, it has become necessary to implement new educational programs enhancing the qualifications of the personnel. However, if the actions taken in the field of education are to be effective, accurate assessment of the competencies and knowledge of nurses and midwives is necessary. For this purpose, the authors of this study attempted to develop and validate a linguistic adaptation (Polish) of the English EBP^2^Q (originally developed in Australia) to assess EBP in Poland [16]. This is the first standardized questionnaire in Poland, allowing us to comprehensively assess the knowledge, attitude, and behavior of students and health care professionals (including nurses and midwives) in terms of EBP.

As the Polish EBP^2^Q is intended for use in a diverse group of nurses and midwives, as well as among students in these fields, a heterogeneous validation sample was chosen. We assume this provides adequate representation for evaluation of the psychometric parameters of the EBP^2^Q and its standardization across the various groups [33]. Our psychometric analysis confirmed the strong performance of the EBP^2^Q. The reliability of the individual domains clearly exceeded the recommended value (Cronbach’s alpha ranged from 0.800 to 0.972) [25, 26]. Our results are similar to those obtained during the validation of the English EBP^2^Q, in which Cronbach’s alpha ranged from 0.760 to 0.940 [16]. The high reliability of the Polish EBP^2^Q contributes to a high degree of confidence in the measurement results obtained. This is linked to the low level of random errors, which do not exceed 10–20%. Thus, random fluctuations in test results described in the classical theory of psychometric tests do not reduce the value of alpha in this case [34].

The results of the test-retest reliability analysis were also similar to those obtained during the validation of the original version of the EBP^2^Q. Both the values of the ICCs (the Polish version: 0.68–0.97 versus the English version: 0.77–0.94) and the differences between the means (the Polish version: 0.42–1.50 versus the English version:−1.07–0.52) for the repeated measurements in individual domains of the EBP^2^Q were comparable to the results of the test-retest analysis published by McEvoy et al. [16]. The results for some statements making up the domains were slightly worse. Slightly lower values were observed for the lower limits of the range of weighted kappas compared with the values for the original version of the questionnaire. However, due to the lack of detailed data from the test-retest analysis performed by McEvoy et al. [16], it is hard to assess whether the lower repeatability (weighted kappas ~0.40) for some statements in both validation tests concerned the same items. To summarize the assessment of the test-retest reliability, the Polish version of the EBP^2^Q appears to have good parameters of absolute stability. This means that the results of the measurements in the individual domains of the EBP^2^Q show limited sensitivity to random changes [35].

Measurement results for the individual statements of the EBP^2^Q obtained during the psychometric evaluation indicate a fairly high degree of similarity in the scope of tested knowledge and attitudes among study participants. We found a small degree of variation in the validation group, which indicates high uniformity in measured traits (SD ranged from 0.81 to 1.38). These results are similar to those obtained by McEvoy et al. [16]. Similar results were also observed in relation to the mean score obtained by respondents for individual items of the questionnaire, and the absence of a “floor” and “ceiling” effect for the validation of the EBP^2^Q (extreme mean values for the English version were 1.71 and 4.09 versus 1.63 and 4.11 for the Polish version) (see Additional file 2 “floor and ceiling effect”).

The assumptions for the validity assessment by factor analysis were the same as those applied in the validation of the English version of the questionnaire [16]. Both the results of Bartlett’s test of sphericity (*P* < 0.0001) and the degree of expected reduction KMO index for the English version 0.92 versus 0.95 for the Polish version) were similar in both studies [16]. The domain structure of the Polish EBP^2^Q was found to be largely consistent with the validated English version. We only observed variability in domains I and II (Relevance and Sympathy). This is probably related to a different organization of the work environment and a slightly different scope of competencies of nurses in Poland and Australia. Generally, despite some differences, the results of analysis of the theoretical validity confirm a high degree of accordance between the planned Polish and validated English EBP^2^Q in evaluating the knowledge and attitudes toward EBP.

The theoretical validity of the Polish EBP^2^Q also confirms the high quality of the measurement tool. Results of respondents, calculated as the sum of the scores obtained for the individual domains, were positively correlated with each other. The exception was domain II (Sympathy), which is associated with the design of the items constituting this domain. In particular, some of the items within domain II directly overlap with those found in domain I (Relevance) and were similar to those found in all other domains. It can therefore be assumed that the individual intercorrelations for the EBP^2^Q components confirm the fulfillment of the condition of theoretical validity. Therefore, some theoretical assumptions related to the construction of the EBP^2^Q were confirmed by empirical observation, and the functioning of the Polish version is similar to the English EBP^2^Q.

Regarding the assessment of discriminative validity, the validation tests slightly differed across studies in how they were performed. For the original version of the EBP^2^Q, McEvoy et al. [16] proposed comparing the scores obtained in three different groups of respondents. The control group was compared with two groups differing in duration and form of EBP training. In the presented Polish validation of the EBP^2^Q, two groups were compared (the “no formal training” control group versus the e-learning group). For both tests, significant differences between the groups concerned the results of the measurements in domains I and III (Relevance and Terminology). As expected, both tests also showed no significant training effect on the results achieved in domain IV (Practice). The differences in the assessment of discriminative validity relating to domain V (Confidence) may be connected with different durations of EBP training in the validation tests. The method of conducting the EBP training is crucial to its effectiveness. Therefore, variations in training programs, including their duration (the Polish version: 12 h versus the English version: ≥20 h) and form (the Polish version: e-learning versus the English version: EBP course as part of university education), may have significantly contributed to the observed differences.

The last element of psychometric assessment of the Polish EBP^2^Q was evaluation of unidimensionality of individual domains. As in the case of validity analysis, these results confirm the factor homogeneity of all the domains, with the exception of domains I and II. In particular, domain II was highly heterogeneous, which directly refers to the results of the evaluation of criterion validity parameter. As mentioned above, this may result from potential differences between Poland and Australia in terms of working conditions and professional competence of nurses or midwives.

The unambiguous results of the psychometric analysis of the Polish version of the EBP^2^Q allow us to assume that it can be used in practice as a tool for assessing the EBP-related competence of students and practitioners in the nursing or midwifery profession. The EBP^2^Q is a tool for measuring the knowledge, attitudes, and behavior of subjects in the field of EBP. The results of this assessment can be used to diagnose the barriers that hinder the implementation of EBP in the workplace. Moreover, the EBP^2^Q can be successfully used by the participants of courses to upgrade their skills in the field of EBP, both as an element of self-assessment, as well as a tool for measuring learning outcomes. The questionnaire can be also used when designing individual educational programs, indicating those areas of knowledge and skills to which particular attention should be paid in the learning process. Furthermore, the modular nature of the EBP^2^Q is its undoubted advantage. In particular, the tool can be adjusted to the needs of an individual test by using only selected domains.

The EBP^2^Q, as any tool, has some drawbacks. One of them is the impossibility to assess a subject’s use of other types of evidence than scientific. Making clinical decisions on the basis of non-scientific evidence, personal experience, tradition or intuition is contrary to the EBP concept. The knowledge of subjects’ attitudes to the use of non-scientific evidence in medical practice could boost the measurement validity. Another important drawback of the EBP^2^Q pertains to domain III (Terminology). This domain relates to the knowledge of certain terms connected with methodology of scientific research, but it includes only the knowledge around quantitative based terms and excludes terms connected with qualitative research.

One of the significant limitations of the present study is the lack of convergent validity analysis, it was not possible to determine this psychometric property due to the lack of another Polish tool available for evaluating similar domains to the EBP^2^Q. In addition, further validation studies should assess the diagnostic ability of the Polish EBP^2^Q, by performing research using the tool in educational situations (i.e., pre- and post-test).

## Conclusions

The results of the psychometric analysis of the Polish EBP^2^Q confirm the high quality of this tool. In terms of reliability and validity, the Polish EBP^2^Q is comparable to the original English version. The questionnaire can be used both in educational activities (graduate and postgraduate studies), as well as an assessment tool among practicing professional nurses and midwives at various levels.

## Additional files

Additional file 1: Evidence-Based Practice Profile (EBP2) Questionnaire. Polska wersja językowa kwestionariusza. (PDF 830 kb)
Additional file 2: Data analysis. (XLSX 26 kb)

## Abbreviations

EBP: Evidence-based practice
EBP^2^Q: Evidence-based practice profile questionnaire
ICC: Intra-class correlation coefficient
KMO: Kaiser-Meyer-Olkin
