# Supplementary material for: Validation study of the Polish version of the Evidence-Based Practice Profile Questionnaire
Source: BMC Med Educ. 2017 Feb 10;17:38. doi: 10.1186/s12909-017-0877-4 (PMC5301392; doi:10.1186/s12909-017-0877-4)
Supplement: Additional file 1: — Evidence-Based Practice Profile (EBP2) Questionnaire. Polska wersja językowa kwestionariusza. (PDF 830 kb) [file 12909_2017_877_MOESM1_ESM.pdf]

Warszawski Uniwersytet Medyczny

Polska wersja językowa (*ver 1.0*)

Evidence-Based Practice Profile (EBP^2) Questionnaire

Celem ankiety jest zebranie danych na temat wiedzy, zachowań i postaw specjalistów ochrony zdrowia wobec praktyki zawodowej opartej na dowodach naukowych

Instrukcja wypełniania

Wypełnienie ankiety zajmuje około 10-12 minut.

Proszę zakreślić jedną cyfrę w każdym wierszu.

Możesz także podać swój komentarz zamieszczając go w polu pod każdą tabelą z pytaniami.

Będziemy bardzo wdzięczni za poświęcenie czasu na wypełnienie niniejszej ankiety.

Oryginalna wersja *Evidence-Based Practice Profile Questionnaire*
© UNIVERSITY OF SOUTH AUSTRALIA McEvoy MP, Williams MT, and Olds TS, Development and psychometric testing of a trans-professional evidence-based practice profile questionnaire. Medical Teacher, 2010; 32(9): e373-80.

Proszę ocenić trafność poszczególnych stwierdzeń w skali 1-5:

		**zdecydowanie się nie zgadzam**	**nie zgadzam się**	**nie mam zdania**	**zgadzam się**	**zdecydowanie się zgadzam**
1.	Znam znaczenie terminu Evidence – based Practice (EBP)	1	2	3	4	5
2.	Mam świadomość istnienia EBP w moim zawodzie	1	2	3	4	5
3.	EBP stanowi nowoczesny model wykonywania mojego zawodu	1	2	3	4	5
4.	Mam świadomość ciągłego rozwoju EBP w moim zawodzie	1	2	3	4	5

Czy ma Pani / Pan dodatkowe komentarze dotyczące udzielonych odpowiedzi?

………

Proszę ocenić trafność poszczególnych stwierdzeń w skali 1-5:

		**z pewnością nie**	**mało prawdopodobne**	**rozważę taką możliwość**	**bardzo prawdopodobne**	**z pewnością tak**
5.	Zamierzam poszerzać własną wiedzę na temat EBP	1	2	3	4	5
6.	Zamierzam rozwijać własne umiejętności w zakresie dostępu i oceny dowodów naukowych istotnych dla mojej praktyki zawodowej	1	2	3	4	5
7.	Zamierzam korzystać z odpowiedniej literatury naukowej w celu aktualizacji posiadanej wiedzy	1	2	3	4	5
8.	Zamierzam zastosować najlepsze dostępne dowody naukowe w celu poprawy jakości własnej praktyki zawodowej	1	2	3	4	5

Czy ma Pani / Pan dodatkowe komentarze dotyczące udzielonych odpowiedzi?

………

Oryginalna wersja *Evidence-Based Practice Profile Questionnaire*
© UNIVERSITY OF SOUTH AUSTRALIA McEvoy MP, Williams MT, and Olds TS, Development and psychometric testing of a trans-professional evidence-based practice profile questionnaire. Medical Teacher, 2010; 32(9): e373-80.

Proszę ocenić trafność poszczególnych stwierdzeń w skali 1-5:

		zdecydowanie się nie zgadzam	nie zgadzam się	nie mam zdania	zgadzam się	zdecydowanie się zgadzam
9.	Zastosowanie EBP w mojej praktyce zawodowej jest niezbędne	1	2	3	4	5
10.	Doniesienia naukowe są przydatne w mojej codziennej praktyce zawodowej	1	2	3	4	5
11.	Powinnam / powinienem częściej wykorzystywać dowody naukowe w mojej codziennej praktyce zawodowej	1	2	3	4	5
12.	Jestem zainteresowana / zainteresowany poprawą umiejętności niezbędnych do włączenia EBP do mojej praktyki zawodowej	1	2	3	4	5
13.	Zastosowanie EBP poprawia jakość mojej praktyki zawodowej	1	2	3	4	5
14.	Zastosowanie EBP pomaga mi podejmować właściwe decyzje kliniczne dotyczące pacjentów podczas mojej praktyki zawodowej	1	2	3	4	5
15.	W mojej codziennej praktyce zawodowej niektóre wymogi EBP mogą mieć ograniczone zastosowanie ze względu np. na brak sprzętu, procedur, personelu, itd.	1	2	3	4	5
16.	Zastosowanie EBP w mojej codziennej praktyce zawodowej nie ma sensu z powodu braku dowodów naukowych na poparcie skuteczności większości wykonywanych przeze mnie czynności zawodowych	1	2	3	4	5
17.	Zastosowanie EBP nie uwzględnia preferencji moich pacjentów	1	2	3	4	5
18.	W podejmowaniu właściwych decyzji w mojej praktyce zawodowej wyżej cenię doświadczenie kliniczne niż wyniki badań naukowych	1	2	3	4	5

Oryginalna wersja *Evidence-Based Practice Profile Questionnaire*
© UNIVERSITY OF SOUTH AUSTRALIA McEvoy MP, Williams MT, and Olds TS, Development and psychometric testing of a trans-professional evidence-based practice profile questionnaire. Medical Teacher, 2010; 32(9): e373-80.

19.	Doświadczenie kliniczne jest najlepszym sposobem oceny skuteczności danego działania	1	2	3	4	5
20.	Krytyczny przegląd piśmiennictwa naukowego i jego znaczenie dla skuteczności leczenia/opieki nad pacjentami nie ma zastosowania w praktyce zawodowej	1	2	3	4	5
21.	Poszukiwanie istotnych dowodów naukowych nie ma zastosowania w praktyce zawodowej	1	2	3	4	5

Czy ma Pani / Pan dodatkowe komentarze dotyczące udzielonych odpowiedzi?

……

Proszę ocenić stopień zrozumienia poszczególnych pojęć:

		**nigdy o nim nie słyszałam**	**słyszałam o nim, jednak nie znam jego znaczenia**	**częściowo rozumiem**	**całkiem dobrze rozumiem**	**rozumiem i potrafię wytłumaczyć innym jego znaczenie**
22.	Ryzyko względne (*RR – Relative risk)*	1	2	3	4	5
23.	Ryzyko bezwzględne *(AR – Absolute risk)*	1	2	3	4	5
24.	Przegląd systematyczny *(Systematic review)*	1	2	3	4	5
25.	Iloraz szans *(OR – Odds ratio)*	1	2	3	4	5
26.	Metaanaliza *(Meta- analysis)*	1	2	3	4	5
27.	*NNT - number needed to treat*	1	2	3	4	5
28.	Przedział ufności *(CI – Confidence interval)*	1	2	3	4	5
29.	Złudzenie publikacyjne *(Publication bias)*	1	2	3	4	5
30.	Wykres leśny *(Forest plot)*	1	2	3	4	5
31.	Zamiar leczenia *(Intention to treat)*	1	2	3	4	5
32.	Istotność statystyczna *(Statistical significance)*	1	2	3	4	5

Oryginalna wersja *Evidence-Based Practice Profile Questionnaire*
© UNIVERSITY OF SOUTH AUSTRALIA McEvoy MP, Williams MT, and Olds TS, Development and psychometric testing of a trans-professional evidence-based practice profile questionnaire. Medical Teacher, 2010; 32(9): e373-80.

33.	*Minimum clinically worthwhile effect*	1	2	3	4	5
34.	Znaczenie kliniczne *(Clinical importance)*	1	2	3	4	5
35.	Randomizowane badanie kliniczne *(RCT – Randomised controlled trial)*	1	2	3	4	5
36.	Wyniki dychotomiczne *(Dichotomous outcomes)*	1	2	3	4	5
37.	Wyniki ciągłe *(Continuous outcomes)*	1	2	3	4	5
38.	Wielkość efektu leczenia *(Treatment effect size)*	1	2	3	4	5

Czy ma Pani / Pan dodatkowe komentarze dotyczące udzielonych odpowiedzi?

………

Jak często w ubiegłym roku:

		**nigdy**	**raz w miesiącu lub rzadziej**	**raz na dwa tygodnie**	**raz na tydzień**	**codziennie**
39.	Sformułowała Pani / sformułował Pan poprawne pytanie kliniczne dotyczące pacjenta, problemu, podjętych działań i ich wyników?	1	2	3	4	5
40.	Znalazła Pani / znalazł Pan dowody naukowe adekwatne do postawionego pytania?	1	2	3	4	5
41.	Korzystała Pani / korzystał Pan z elektronicznej bazy piśmiennictwa naukowego?	1	2	3	4	5
42.	Krytycznie oceniła Pani / ocenił Pan poprawność metodologiczną wykorzystanej literatury naukowej?	1	2	3	4	5
43.	Odniosła Pani / odniósł Pan wyniki badań naukowych do własnej diagnozy?	1	2	3	4	5
44.	Uwzględniała Pani / uwzględniał Pan preferencje pacjenta przy podejmowaniu decyzji klinicznych?	1	2	3	4	5

Oryginalna wersja *Evidence-Based Practice Profile Questionnaire*
© UNIVERSITY OF SOUTH AUSTRALIA McEvoy MP, Williams MT, and Olds TS, Development and psychometric testing of a trans-professional evidence-based practice profile questionnaire. Medical Teacher, 2010; 32(9): e373-80.

45.	Czytała Pani / czytał Pan wyniki opublikowanych badań naukowych?	1	2	3	4	5
46.	Nieformalnie omawiała Pani / omawiał Pan doniesienia naukowe ze współpracownikami?	1	2	3	4	5
47.	Formalnie omawiała Pani / omawiał Pan doniesienia naukowe podczas posiedzeń naukowych?	1	2	3	4	5

Czy ma Pani / Pan dodatkowe komentarze dotyczące udzielonych odpowiedzi?

……

Proszę ocenić poziom umiejętności związanych z EBP:

		**zdecydowanie nie umiem / nie potrafię**	**nie umiem / nie potrafię**	**nie mam zdania**	**umiem, potrafię**	**zdecydowanie umiem/ potrafię**
48.	Umiejętności badawcze	1	2	3	4	5
49.	Obsługa komputera	1	2	3	4	5
50.	Zdolność do identyfikacji braków w wiedzy	1	2	3	4	5
51.	Zdolność do formułowania poprawnych pytań klinicznych	1	2	3	4	5
52.	Znajomość głównych typów informacji i ich źródeł	1	2	3	4	5
53.	Umiejętność wyszukiwania informacji w elektronicznych bazach piśmiennictwa naukowego	1	2	3	4	5
54.	Umiejętność uzyskania dostępu do dowodów naukowych (uzyskanie kopii artykułów lub raportów)	1	2	3	4	5
55.	Umiejętność krytycznej analizy dowodów naukowych w świetle stosowanych norm	1	2	3	4	5
56.	Umiejętność określenia stopnia wiarygodności uzyskanego dowodu naukowego	1	2	3	4	5
57.	Zdolność do określenia stopnia użyteczności klinicznej uzyskanego dowodu naukowego	1	2	3	4	5

Oryginalna wersja *Evidence-Based Practice Profile Questionnaire*
© UNIVERSITY OF SOUTH AUSTRALIA McEvoy MP, Williams MT, and Olds TS, Development and psychometric testing of a trans-professional evidence-based practice profile questionnaire. Medical Teacher, 2010; 32(9): e373-80.

58.	Umiejętność zastosowania informacji naukowej do poszczególnych przypadków (tj. zintegrowanie wyników badań z osobistymi preferencjami, wartościami, obawami i oczekiwaniami własnymi i pacjenta)	1	2	3	4	5

Czy ma Pani / Pan dodatkowe komentarze dotyczące udzielonych odpowiedzi?

………

Proszę ocenić trafność poszczególnych twierdzeń w skali od 1 do 5:

		**zdecydowanie się nie zgadzam**	**nie zgadzam się**	**nie mam zdania**	**zgadzam się**	**zdecydowanie się zgadzam**
59.	Chcę zdobywać nowe informacje	1	2	3	4	5
60.	Krytycznie oceniam nowe pomysły	1	2	3	4	5
61.	Mam predyspozycje do zarządzania	1	2	3	4	5
62.	W rozwiązywaniu problemów posługuję się planem	1	2	3	4	5
63.	Lubię się uczyć	1	2	3	4	5
64.	W mojej pracy zawodowej kadra zarządzająca stale poszukuje nowych możliwości uczenia się	1	2	3	4	5
65.	Znajduję czas na czytanie badań naukowych	1	2	3	4	5
66.	Brak czasu jest jedną z największych barier uniemożliwiających wykorzystywanie EBP w mojej praktyce zawodowej	1	2	3	4	5
67.	Obciążenie pracą zawodową uniemożliwia mi regularną aktualizację mojej wiedzy	1	2	3	4	5
68.	Koszty użytkowania zasobów informacyjnych ograniczają zastosowanie EBP w praktyce zawodowej	1	2	3	4	5
69.	Dostęp do komputera ma wpływ na zastosowanie EBP w mojej praktyce zawodowej	1	2	3	4	5
70.	Wykorzystanie dostępnych źródeł wiedzy wystarcza do stosowania EBP w mojej praktyce zawodowej	1	2	3	4	5

Oryginalna wersja *Evidence-Based Practice Profile Questionnaire*
© UNIVERSITY OF SOUTH AUSTRALIA McEvoy MP, Williams MT, and Olds TS, Development and psychometric testing of a trans-professional evidence-based practice profile questionnaire. Medical Teacher, 2010; 32(9): e373-80.

71.	Wsparcie współpracowników jest jedną z największych motywacji do stosowania EBP w praktyce zawodowej	1	2	3	4	5
72.	Wsparcie ze strony kadry zarządzającej jest jedną z największych motywacji do stosowania EBP w praktyce zawodowej	1	2	3	4	5
73.	Mój pracodawca wymaga ode mnie używania EBP w codziennej praktyce zawodowej	1	2	3	4	5
74.	Mam już dosyć EBP	1	2	3	4	5

Czy ma Pani / Pan dodatkowe komentarze dotyczące udzielonych odpowiedzi?

……

Oryginalna wersja *Evidence-Based Practice Profile Questionnaire*
© UNIVERSITY OF SOUTH AUSTRALIA McEvoy MP, Williams MT, and Olds TS, Development and psychometric testing of a trans-professional evidence-based practice profile questionnaire. Medical Teacher, 2010; 32(9): e373-80.

Uwagi dotyczące posługiwania się skalą EBP^2

Kwestionariusz EBP^2 jest sześciomodułowy, moduły można stosować niezależnie jako oddziałane ankiety w zależności od potrzeb danego badania.

Podskala	Opis domeny	Pozycje skali
**I**	Stosunek wobec poszerzania własnych kompetencji dotyczących *Evidence-based Practice*	1-14
**II**	Postawa wobec wybranych aspektów *Evidence-based Practice* w pracy zawodowej	15-21
**III**	Wiedza na temat znajomości terminologii związanej z badaniami naukowymi	22-38
**IV**	Częstości wykorzystywania poszczególnych elementów *Evidence-based Practice* w codziennej pracy klinicznej	39-47
**V**	Umiejętności związanych z *Evidence-based Practice*	48-58
**VI**	Pozostałe aspekty związane z *Evidence-based Practice*	59-74

Wszystkie podskale z wyjątkiem II nie wymagają rekodowania, a wynik pomiaru jest sumą punktów uzyskaną przez respondenta z danego modułu kwestionariusza.

Podskala II z wyłączeniem pozycji 15 jest kodowana odwrotnie i przed jej interpretacją należy wyniki rekodować przyznając następującą punktację w zależności od wskazań respondenta: 1 (5 pkt.), 2 (4 pkt.), 3 (3 pkt.), 4 (2 pkt.) i 5 (1 pkt.). Po zsumowaniu wyników dla pozycji od 15 do 21 uzyskujemy właściwy wynik dla podskali II „Postawa wobec wybranych aspektów *Evidence-based Practice* w pracy zawodowej”.

Interpretacja wyników pomiaru: im wyższy wynik tym wyższe natężenie poszczególnych zmiennych. Z uwagi na uzyskanie sześciu odrębnych wyników dla każdej osoby badanej możliwa jest zarówno analiza oddzielnych wyników, jak i analiza uzyskanego profilu zmiennych. Nie wyznaczono norm dla poszczególnych podskal.

Oryginalna wersja *Evidence-Based Practice Profile Questionnaire*
© UNIVERSITY OF SOUTH AUSTRALIA McEvoy MP, Williams MT, and Olds TS, Development and psychometric testing of a trans-professional evidence-based practice profile questionnaire. Medical Teacher, 2010; 32(9): e373-80.
